# Understanding the relationships between parenting, attachment, schemas and psychosis: A serial mediation analysis

**DOI:** 10.1111/bjc.12545

**Published:** 2025-05-10

**Authors:** Nadia Akers, Christopher D. J. Taylor, Katherine Berry

**Affiliations:** ^1^ Division of Psychology and Mental Health, School of Health Sciences, Faculty of Biology, Medicine and Health, Manchester Academic Health Sciences Centre The University of Manchester Manchester UK; ^2^ Research and Innovation Greater Manchester Mental Health NHS Foundation Trust, Manchester Royal Infirmary Manchester UK; ^3^ Pennine Care NHS Foundation Trust School of Psychology, Faculty of Science, The University of Sheffield Ashton Under Lyne UK; ^4^ School of Psychology, Faculty of Science The University of Sheffield Sheffield UK

**Keywords:** attachment, parenting, psychosis, schemas

## Abstract

**Background:**

Parenting experiences during childhood have been suggested to inform the development of an individual's attachment style and core schemas. Additionally, parenting, attachment and schemas have all individually been linked to symptoms of psychosis in adulthood. However, there is limited research drawing together these concepts to understand the process by which parenting influences psychosis in adulthood. The current study, therefore, aimed to test a serial mediation model in which attachment and schemas mediate the relationship between parenting experiences and adult experiences of psychosis.

**Method:**

A cross‐sectional study collected data from 132 adult participants regarding their childhood caregivers' parenting style, their attachment style, core schemas, and adult symptoms of psychosis.

**Results:**

A serial mediation analysis revealed that the relationship between abusive or overcontrolling parenting and psychosis in adulthood was fully mediated by anxious or disorganized attachment styles and negative schema. The relationship between indifferent parenting and psychosis was fully mediated by avoidant attachment and negative schemas.

**Conclusions:**

The findings support the tested hypotheses suggesting that attachment and schemas act as serial mediators in the relationship between parenting style and psychosis. The results highlight the importance of considering attachment and schemas when working therapeutically with people with psychosis. Further research is needed to elaborate on this understanding, develop early parenting interventions to support parents to foster secure attachment in their children and place a focus on schema change within CBT for psychosis.

## INTRODUCTION

Psychosis is characterized by significant shifts in an individual's perception, mood, thoughts and behaviour. People may present with positive symptoms such as hallucinations and delusions, and/or negative symptoms such as apathy, social withdrawal and reduced speed (National Institute for Health and Care Excellence, 2014). The role of social and family environments, childhood adversity and trauma in psychosis is well researched (Beyer et al., 2024; Varese et al., 2012). However, research often conflates trauma within and outside of the home environment. Physical, sexual or emotional abuse from parents within the home environment could be considered to have a greater detrimental effect due to the lack of a child's safe place. There is a dearth of research examining more subtle acts of parenting and psychosis, which may not be captured by childhood adversity and trauma research. For example, although styles of parenting such as overcontrol and criticism from early caregivers are hypothesized to influence the risk of an individual's later development of psychosis, the process by which this occurs is not fully understood (Mansueto et al., 2018).

One potential mediator in the relationship between early parenting experiences and the development of mental health problems later in life is attachment style; an individual's interpersonal strategy of relating to significant others (Bowlby, 1969). A secure attachment style is hypothesized to develop as a result of responsive and warm parenting styles; thus, children grow up perceiving their caregivers as a safe and secure base (Ainsworth et al., 1978; Hazan & Shaver, 1986). If care is not sufficiently responsive and warm, an individual either develops an avoidant or anxious attachment pattern which are referred to as insecure attachment styles. Avoidant attachment has been associated with indifferent or neglectful parenting and is characterized by the avoidance of closeness with others and difficulty expressing emotions (Bartholomew & Horowitz, 1991). Anxious attachment has been linked to inconsistent or overly intrusive parenting and is characterized by excessive reassurance seeking and fear of abandonment within relationships (Bartholomew & Horowitz, 1991). A fourth pattern, known as disorganized attachment style, is hypothesized to develop as a result of significant abuse during childhood, including physical and sexual abuse, and is characterized by unpredictable patterns of behaviour within relationships and a lack of a coherent strategy in terms of regulating distress (Main & Solomon, 1986).

In the context of psychosis, individuals with secure attachment styles have been found to have lower rates of delusions and hallucinations (Bucci et al., 2017) and avoidant, anxious and disorganized attachment been all been associated higher rates of psychotic experiences (Carr et al., 2018; Gumley et al., 2014). By virtue of the fact that attachment styles first develop in within early caregiving relationships, we could hypothesize that parenting may shape an individual's future attachment style and subsequently impact on future psychotic symptoms. In support of this hypothesis, insecure attachment styles have been found to mediate the relationship between traumatic childhood experiences and adult experiences of psychosis. For example, Pilton et al. (2016) found that anxious attachment mediated the relationship between the experience of abuse and neglect in childhood and the severity and distress caused by hearing voices. Additionally, disorganized attachment has been seen to mediate the relationship between childhood trauma and negative symptoms of psychosis (Degnan et al., 2022). In the same study, avoidant attachment did not mediate the relationship between trauma and negative symptoms, which the authors attributed to the fact avoidant attachment styles were associated with neglectful parenting as opposed to overt trauma.

A second potential mediator of the relationship between parenting styles and the development of mental health problems later in life is schemas, which are core beliefs that an individual holds about themselves and others, as a result of their past experiences (Beck, 1979). Positive schemas in adulthood have been associated with positive perceptions of parents consistently meeting core emotional needs (Louis et al., 2020), whereas negative, or maladaptive schemas have been associated with experiences of neglectful, abusive, inconsistent or overprotective parenting (Bruysters & Pilkington, 2023; Wearden et al., 2008). Maladaptive schemas about the self and others are suggested to contribute to the development and severity of psychosis (Fowler et al., 2006; Taylor & Harper, 2017). Research further suggests that the development of schemas may be influenced by an individual's early attachment style (Simard et al., 2011; Young et al., 2003). This is supported by a meta‐analysis by Karantzas et al. (2023), in which a relationship was found between early maladaptive schemas and attachment style, demonstrating a significant positive association between insecure attachment styles and maladaptive schemas. This indicates that it may not be the parenting style itself, but the resulting attachment style which contributes to the development of negative schemas. It could therefore be hypothesized that attachment style influences adult symptoms of psychosis, through negative schematic beliefs. In support of this hypothesis, negative other schemas are shown to mediate the relationship between disorganized attachment and paranoia in psychosis (Humphrey et al., 2022). Additionally, two systematic reviews have highlighted negative beliefs about the self and others as positive mediators in the relationship between attachment and psychotic symptoms, in psychosis samples and in samples with non‐clinical psychosis experiences (Partridge et al., 2022; Sood et al., 2022). Cross‐sectional mediation studies have also suggested that insecure attachment was associated with higher negative self‐beliefs and subsequently higher levels of paranoia (Martinez et al., 2021; Pickering et al., 2008; Udachina & Bentall, 2014). Furthermore, using path analysis, Humphrey et al. (2022) found sequential links between childhood interpersonal trauma, disorganized attachment, negative self and other schema and experiences of paranoia in people with psychosis.

Drawing together research on parenting, attachment and schemas indicates there may be a sequential relationship from parenting style to psychosis in which attachment style and schemas are mediating factors. Attachment styles developed through interactions with caregivers in early childhood may contribute to the development of maladaptive core self and other schemas, subsequently influencing the development and severity of psychosis symptoms. However, this sequential process has not been directly investigated and although there is robust evidence regarding the role of trauma in psychosis, there is limited focus within the literature on childhood adversity related to a more subtle consideration of parenting styles. Increasing understanding about the role of parenting, attachment and schemas in psychosis will inform both theory and practice, supporting the continual development and refinement of psychological therapies for psychosis. Consequently, the aim of the current study is to provide a preliminary assessment of this model. It was hypothesized that warm and responsive parenting would be negatively correlated with all avoidant, anxious and disorganized attachment styles, and in a serial, causal order, participants' insecure attachment style and negative schemas would positively mediate the relationship between their experiences of their caregivers' parenting style during childhood and their symptoms of psychosis in adulthood. It was further hypothesized that anxious and disorganized attachment styles would act positively as serial mediators when parenting was abusive or overcontrolling, whereas avoidant attachment style would only present as a positive mediator when parental style was indifferent, assuming that indifferent parenting is an indicator of neglect.

## METHOD

### Design and sample

An online cross‐sectional study was conducted. Participants were eligible to participate if they: (1) self‐reported that they were aged 18 or over; (2) self‐reported having received either: a diagnosis of schizophrenia, schizoaffective or schizophreniform disorder, delusional disorder, brief psychotic disorder, affective psychosis (including bipolar or depressive disorders with psychotic features) or any other disorder which included psychotic experiences, and/or received antipsychotic medication and/or support from mental health services for their psychotic experiences; and (3) self‐reported that they were fluent in English. These criteria have been used in other online studies of psychosis (Degnan et al., 2022; Humphrey et al., 2022). A Monte Carlo power analysis for indirect effects indicated that a sample size of 140 would be sufficient to achieve 80% power (Schoemann et al., 2017).

### Measures

#### Sociodemographic and clinical information

Participants provided information regarding demographics and their primary caregiver(s) during the first 18 years of their life. Information regarding diagnosis and treatment was also collected to assess eligibility for the study.

#### Independent variables

The Measure of Parenting Style (MOPs; Parker et al., 1997) was used to measure parenting style. It is a 15‐item scale in which participants rate the truth of each statement of parental maltreatment on a 4‐point Likert scale, ranging from not at all (0) to extremely (3), with subscale scores (indifference, abuse, overcontrol) calculated for both maternal and paternal parenting. The MOPS has been shown to have good internal consistency and concurrent validity (Parker et al., 1997). The internal consistency of the abuse subscale of the MOPS in the current study was excellent (*α* = .90) and the indifference subscale was acceptable (.88). However, the internal consistency of the overcontrol subscale was questionable, at .68. The Parental Care‐giving Style Questionnaire (PCSQ; Hazan & Shaver, 1986) was also used to measure parenting. It is a 6‐item measure asking participants to rate three paragraphs on a 5‐point Likert scale based on similarity to their parent(s) style of parenting, ranging from strongly disagree (1) to strongly agree (5). Both measures were selected to assess parenting, as although the MOPS is a more comprehensive measure of parental maltreatment, the PCSQ includes a positive item for warm and responsive parenting. Participants were encouraged to complete the measure with regard to the caregiver(s) whom they had the most contact with during the first 18 years of life, if not their mother and father.

#### Mediators

The Psychosis Attachment Measure–Revised (PAMR; Pollard et al., 2020) was used to measure attachment style. It is a 23‐item measure including a subscale of disorganized attachment. Participants were required to rate each item statement on a 4‐point Likert scale ranging from not at all (0) to very much (3), resulting in three subscales: anxious, avoidant and disorganized. The PAMR has been shown to have good test–retest reliability, internal consistency and construct validity (Justo‐Nunez et al., 2024; Pollard et al., 2020). The internal consistency of the avoidant subscale in the current study was acceptable (*α* = .78), the anxious subscale was good (*α* = .84), and the disorganized subscale was excellent (*α* = .90).

The Brief Core Schema Scales (BCSS; Fowler et al., 2006) was used to measure positive and negative schema about the self and others. The 24‐item scale is designed for use with participants with experience of psychosis. Participants rated the strength of each belief on a 4‐point Likert scale, ranging from ‘believe it slightly’ (1) to ‘believe it totally’ (4). The BCSS has been shown to have excellent test–retest reliability and good internal consistency and has demonstrated concurrent and discriminant validity (Fowler et al., 2006; Gracie et al., 2007; Hardy et al., 2016). In the current study, the negative self subscale demonstrated acceptable internal consistency (*α* = .76), as did the positive self subscale (*α* = .78). The negative other and positive other subscales demonstrated good internal consistency (*α* = .86 and .85, respectively).

#### Dependent variable

The Community Assessment of Psychic Experiences (CAPE; Stefanis et al., 2002) was used to measure symptoms of psychosis. Participants rated their experience of each item on a 4‐point Likert scale, ranging from never (1) to always (4), and included frequency and distress scores for three symptom domains: positive, negative and depressive. The 42‐item measure has good psychometric validity and reliability in both general population and clinical samples (Konings et al., 2006; Stefanis et al., 2002; Yung et al., 2009). In the current study, the positive frequency (*α* = .89), depressive frequency (*α* = .88), depressive distress (*α* = .88) and negative frequency (*α* = .88) subscales had good internal consistency. The positive distress (*α* = .94) and negative distress (*α* = .94) subscales showed excellent internal consistency. Both frequency and distress scores were collected in the current study for descriptive purposes. However, in cases where frequency and distress scores were highly correlated, only frequency scores were used for mediation analyses.

### Recruitment and procedure

Ethical approval was granted by the University of Manchester Research Ethics Committee. Participants were recruited through advertisements on social media and mental health websites and forums. Local, national and international mental health organizations, peer support groups and charities were contacted via email to request sharing of the study among members and wider networks. After confirming consent to the study, participants were asked to complete an eligibility questionnaire which enquired about their age, fluency in English and any diagnoses or treatment for psychosis. Participants who were eligible were then presented with the sociodemographic questionnaire followed by each of the study measures in the following order: MOPS, PCSQ, PAMR, BCSS and CAPE. Following completion, participants were debriefed with information on relevant mental health support organizations and given the option to take part in a prize draw. The average completion time was 43 min.

### Data analysis

Data were analysed using SPSS statistical software version 29 (IBM Corp, 2023). Descriptive statistics were calculated for demographic and clinical information, in addition to questionnaire data, *Z*‐scores for skewness and kurtosis were calculated to assess the normality of distributions. If *Z*‐values were ± 3.29, the data were considered to be of a normal distribution, in line with recommendations for medium sized (50 ≤ *n* < 300) samples (Kim, 2013; Mishra et al., 2019). Differences on study measures between participants with and without missing measure data were examined using a between‐group analysis. Preliminary analyses included Pearson's correlations and ANOVAs to investigate significant relationships between measure subscales and demographic characteristics, to determine significant variables for inclusion in the mediation models. Bias‐corrected bootstrapping with 5000 random samples was used to correct for any distributions that differed from normality (Cheung & Lau, 2008).

Serial mediation analyses were conducted in SPSS using PROCESS (Model 6) to examine the hypotheses (Hayes, 2017). Serial mediation models (SMM) explored the indirect, direct and total effect of parental abuse/overcontrol ➔ anxious/disorganized/avoidant attachment ➔ negative self/other schema ➔ positive/negative symptoms. Additional SMMs explored the indirect, direct and total effect of parental indifference ➔ avoidant attachment ➔ negative self/other schema ➔ positive/negative symptoms. All models were conducted for both mother and father parental figures. Direct and indirect effects were tested using a bootstrap estimation approach with 5000 samples (Hayes, 2017). The null hypothesis was rejected when the 95% bias‐corrected confidence interval did not include zero. Demographic characteristics which were seen to be significantly related to subscales of the dependent variable (CAPE positive and negative symptom frequency), were included as covariates within supplementary analyses, without a theoretical basis to include them in the main analyses.

## RESULTS

### Sample and descriptive statistics

A total of 178 participants consented to take part in the study. However, only 132 participants completed at least two measures and therefore were included in the analyses below. Missing data was present for 13% of the final sample. Between‐group analyses between participants with and without missing data revealed no significant differences in any demographic characteristics, parenting experiences or attachment style. The sample was relatively young with a median age of 35 years and the majority were White (83%). Mothers were most often the primary caregiver. See Table 1 for sample characteristics.

**TABLE 1:** Demographic and clinical characteristics of the sample (*n* = 132).

Demographic/clinical information		Descriptive statistic
Age	Median (IQR)	35.00 (16)
	Range (years)	19–82
Gender, *n* (%)	Female	88 (66.7%)
	Male	34 (25.8%)
	Non‐binary	10 (7.6%)
Ethnicity, *n* (%)	White British	61 (46.21%)
	White Irish	10 (7.58%)
	White other	39 (29.55%)
	Mixed – White and Black Caribbean	3 (2.27%)
	Mixed – White and Black African	2 (1.51%)
	Black or Black British – African	1 (.76%)
	Mixed – White and Asian	3 (2.27%)
	Asian or Asian British – Indian	4 (3.03%)
	Asian or Asian British – Pakistani	1 (.76%)
	Any other Asian/Asian British	1 (.76%)
	Chinese	1 (.76%)
	Other	6 (4.54%)
	Total	132 (100%)
Marital status, *n* (%)	Never married	92 (69.70%)
	Married	20 (15.15%)
	Registered same‐sex partnership	4 (3.03%)
	Separated	2 (1.52%)
	Divorced	11 (8.33%)
	Total	129 (97.73%)
	Missing	3 (2.27%)
Education level, *n* (%)	No qualifications	11 (8.33%)
	GCSE's or equivalent	10 (7.58%)
	A level equivalent or higher	21 (15.91%)
	Degree level qualification or higher	90 (68.18%)
	Total	132 (100%)
Employment status, *n* (%)	Employed	53 (40.15%)
	Self‐employed	8 (6.06%)
	Voluntary work	1 (.76%)
	Unemployed	17 (12.88%)
	Full‐time education	9 (6.81%)
	Part‐time education	2 (1.52%)
	Looking after family/home	3 (2.27%)
	Sickness or disability benefits	38 (28.79%)
	Retired	1 (.76%)
	Total	132 (100%)
Primary caregiver(s), *n* ^a^	Mother	128
	Father	81
	Grandparent(s)	14
	Aunt or Uncle	4
	Sibling	12
	Foster carer(s)	3
	Adoptive parents	2
	Stepmother	4
	Stepfather	4
	Other	3
Self‐reported diagnoses, *n* ^a^	Schizophrenia/Paranoid Schizophrenia	37
	Schizoaffective Disorder	27
	Depression with psychotic experiences	41
	Delusional Disorder	5
	Bipolar Disorder with psychotic experiences	35
	Brief Psychotic Disorder	13
	Other disorder which included psychotic experiences	38
MOPS, mean (SD)	Maternal indifference	5.67 (4.92)
	Maternal abuse^b^	4.00 (7.00)
	Maternal overcontrol	5.53 (3.38)
	Paternal indifference	6.44 (5.38)
	Paternal abuse	6.02 (5.25)
	Paternal overcontrol	4.38 (3.57)
PCSQ mother, mean (SD)	Warm and responsive	3.28 (2.09)
	Cold and rejecting^b^	4.00 (5.00)
	Inconsistent ambivalent^b^	4.00 (5.00)
PCSQ father, mean (SD)	Warm and responsive	3.17 (2.14)
	Cold and rejecting	3.86 (2.21)
	Inconsistent ambivalent^b^	4.00 (5.00)
PAMR, mean (SD)	Anxious	1.64 (.73)
	Avoidant	1.91 (.65)
	Disorganized	1.47 (.78)
BCSS, mean (SD)	Negative self	8.62 (6.62)
	Negative other^b^	6.00 (10.00)
	Positive self^b^	6.00 (10.00)
	Positive other^b^	5.00 (9.00)
CAPE, mean (SD)	Positive frequency	38.63 (10.71)
	Positive distress	29.72 (15.12)
	Depressive frequency	20.15 (5.59)
	Depressive distress	18.71 (7.53)
	Negative frequency	32.73 (8.74)
	Negative distress	26.85 (10.68)

^a^ Participants could select multiple options.

^b^ In cases where the data were not normally distributed, the mean (SD) was replaced with median (IQR).

### Preliminary analyses

Correlational analyses between subscale measures can be found in Table 2. Consistent with hypotheses, the PCSQ warm and responsive parenting subscale was significantly negatively correlated with avoidant and disorganized attachment. The MOPS subscales were highly correlated with PSCQ cold and rejecting and inconsistent ambivalent subscales; however, the MOPS was more significantly correlated with anxious and disorganized attachment than the PCSQ. Therefore, the MOPS was selected as the parenting style measure for subsequent mediation analyses. All PAMR subscales were correlated with the BCSS negative subscales, with the PAMR anxious and disorganized subscales displaying a stronger correlation than the PAMR avoidant subscale. The CAPE symptom frequency and distress caused by symptoms scores were highly correlated; moreover, symptom frequency subscale scores only were included in the mediation analyses, as study hypotheses concerned the presence of symptoms. ANOVA results revealed significant differences between ethnic groups with regard to the CAPE Positive Symptom Frequency subscale (*p* = .04) and between employment status groups with regards to the CAPE Negative Symptom Frequency subscale (*p* = .01). Thus, ethnicity and employment status were included as covariates within supplementary analyses, and no differences in results were seen on inclusion (Tables A1, A2, A3).

**TABLE 2:** Correlations between measures.

	1	2	3	4	5	6	7	8	9	10	11	12	13	14	15	16	17	18	19	20	21	22	23	24	25
1 MOPS indifference mother	0																								
2 MOPS abuse mother	.62**	0																							
3 MOPS overcontrol mother	.47**	.60**	0																						
4 MOPS indifference father	.50**	.25**	.33**	0																					
5 MOPS abuse father	.24**	.24**	.16	.57**	0																				
6 MOPS overcontrol father	.22**	.06	.11	.39**	.70**	0																			
7 PCSQ mother warm and responsive	−.66**	−.55**	−.57**	−.31**	−.10	−.02	0																		
8 PCSQ mother cold and rejecting	.68**	.50**	.40**	.29**	.01	.02	−.59**	0																	
9 PCSQ mother inconsistent ambivalent	.34**	.53**	.46**	.16	.07	−.02	−.46**	.51**	0																
10 PCSQ father warm and responsive	−.29**	−.17	−.17	−.62**	−.54**	−.39**	.33**	−.10	−.13	0															
11 PCSQ father cold and rejecting	.26**	.22*	.18*	.69**	.41**	.26**	−.17	.24**	.16	−.60**	0														
12 PCSQ father inconsistent ambivalent	.10	−.04	−.05	.36**	.45**	.49**	−.00	−.04	.04	−.40**	.34**	0													
13 PAMR anxious	.22*	.26**	.28**	.23*	.22*	.30**	−.11	.19*	.21*	−.10	.15	.13	0												
14 PAMR avoidant	.25**	.13	.10	.24**	.09	.09	−.21*	.25**	.17	−.07	−.00	.05	.15	0											
15 PAMR disorganized	.38**	.22*	.24**	.39**	.21*	.26**	−.18*	.27**	.20*	−.22*	.26**	.22*	.62**	.43**	0										
16 BCSS negative self	.21*	.21*	.19*	.31**	.23**	.31**	−.07	.16	.16	−.20*	.21*	.19*	.60**	.36**	.57**	0									
17 BCSS positive self	−.08	−.09	−.08	−.22*	−.04	−.02	.10	−.09	−.06	.17	−.13	.02	−.34**	−.27**	−.25**	−.59**	0								
18 BCSS negative others	.28**	.22*	.16	.24**	.20*	.33**	−.45	.20*	.22*	−.17	.11	.20*	.38**	.36**	.50**	.49**	−.08	0							
19 BCSS positive others	−.17	−.20*	−.06	−.25**	−.10	−.08	.17	−.05	−.14	.27**	−.23**	−.08	−.26**	−.49**	−.39**	−.33**	.46**	−.38**	0						
20 CAPE positive frequency	.14	.16	.16	.11	.21*	.16	−.02	.14	.26**	.04	.00	.14	.37**	.23*	.44**	.41**	.02	.56**	−.14	0					
21 CAPE positive distress	.11	.14	.13	.18*	.28**	.20*	−.00	.12	.19*	−.06	.14	.15	.41**	.15	.38**	.47**	−.13	.50**	−.14	.86**	0				
22 CAPE depressive frequency	.33**	.32**	.28**	.40**	.29**	.25**	−.11	.24**	.21*	−.20*	.25**	.22*	.68**	.36**	.56**	.74**	−.47**	.48**	−.32**	.48**	.55**	0			
23 CAPE depressive distress	.27**	.23*	.17	.35**	.27**	.21*	−.10	.21*	.10	−.18	.25**	.18	.62**	.27**	.46**	.60**	−.37**	.38**	−.23*	.36**	.52**	.87**	0		
24 CAPE negative frequency	.21*	.13	.27**	.28**	.20*	.19*	−.09	.24**	.16	−.10	.08	.15	.54**	.41**	.50**	.61**	−.35**	.43**	−.26**	.53**	.57**	.67**	.55**	0	
25 CAPE negative distress	.20*	.02	.13	.38**	.20*	.21*	−.04	.23*	.05	−.15	.21*	.16	.51**	.25	.44**	.51**	−.30**	.30**	−.31	.39**	.57**	.61**	.68**	.77**	0

Abbreviation: BCSS, Brief Core Schema Scales; CAPE, Community Assessment of Psychic Experiences; MOPS, Measure of Parental Style; PAM–R, Psychosis Attachment Measure – Revised; PCSQ, Parental Caregiving Style Questionnaire.

*p < .05, **p < .01, ***p < .001.

### Mediation analyses

Insecure (anxious, disorganized and avoidant) attachment styles and negative (self and other) schemas were entered as mediating variables between (abusive and overcontrolling) parenting style (MOPS) and frequency of (positive and negative) symptoms of psychosis. Insecure avoidant attachment style and negative (self and other) schemas were also entered as mediating variables between indifferent parenting (MOPS) and frequency of (positive and negative) symptoms of psychosis. Including ethnicity and employment status as covariates in supplementary analyses did not result in any significant differences to the results of the serial mediation models below (Tables A1, A2, A3).

#### Abusive/overcontrolling parenting and anxious/disorganized attachment

The results of the serial mediation analyses of anxious/disorganized attachment and negative schema in the relationship between abusive/overcontrolling parenting style and symptom frequency can be found in Tables 3 (maternal) and 4 (paternal). Bootstrap confidence intervals showed a significant positive indirect effect of abusive maternal and paternal parenting style on positive symptom frequency through anxious attachment and negative schemas. This indirect effect was seen for both negative self and negative other schemas. There was also a significant positive indirect effect of abusive maternal and paternal parenting on positive symptom frequency through disorganized attachment and negative self and negative other schemas. A significant positive indirect effect of overcontrolling maternal and paternal parenting style on positive symptom frequency through anxious attachment and negative self and negative other schemas was also observed. Similarly, there was a significant positive indirect effect of overcontrolling maternal and paternal parenting on positive symptom frequency, through disorganized attachment and negative self and negative other schemas. These results were replicated with regard to negative symptom frequency. However, non‐significant results were seen for the direct effect of abusive maternal and paternal parenting style on positive and negative symptom frequency; and for the direct effect of overcontrolling parenting style on positive and negative symptom frequency.

**TABLE 3:** Indirect, direct and total effects for the relationship between maternal abusive and overcontrolling parenting styles and symptom frequency, serially mediated by insecure attachment style and negative schemas (*n* = 115).

Independent variable	Dependent variable	Mediators	Indirect effect (95% CI)	Direct effect (95% CI)	Total effect (95% CI)
MOPS maternal abuse	CAPE positive frequency	PAMR anxious ➔ BCSS Negative self	.10 (.014 to .22)	.13 (−.27 to .53)	.24 (.05 to .49)
		PAMR anxious ➔ BCSS Negative others	.10 (.02 to .21)	.02 (−.34 to .39)	.34 (.10 to .64)
		PAMR disorganized ➔ BCSS Negative self	.07 (.01 to .17)	.10 (−.29 to .49)	.27 (.07 to .53)
		PAMR disorganized ➔ BCSS Negative others	.12 (.03 to .25)	.03 (−.33 to .39)	.33 (.09 to .63)
		PAMR avoidant ➔ BCSS Negative self	.04 (−.01 to .12)	.17 (−.24 to .57)	.20 (.02 to .44)
		PAMR avoidant ➔ BCSS Negative others	.06 (−.02 to .17)	.09 (−.28 to .46)	.28 (.04 to .56)
	CAPE negative frequency	PAMR anxious ➔ BCSS Negative self	.12 (.03 to .24)	−.05 (.71 to −.33)	.30 (.07 to .55)
		PAMR anxious ➔ BCSS Negative others	.04 (.01 to .10)	−.07 (−.36 to .23)	.31 (.11 to .52)
		PAMR disorganized ➔ BCSS Negative self	.12 (.03 to .23)	−.04 (−.32 to .25)	.28 (.07 to .51)
		PAMR disorganized ➔ BCSS Negative others	.05 (.01 to .12)	−.01 (−.32 to .29)	.26 (.08 to .43)
		PAMR avoidant ➔ BCSS Negative self	.05 (−.02 to .13)	−.01 (−.29 to .26)	.26 (.05 to .48)
		PAMR avoidant ➔ BCSS Negative others	.03 (−.01 to .08)	.05 (−.27 to .36)	.20 (.24 to .39)
MOPS maternal overcontrol	CAPE positive frequency	PAMR anxious ➔ BCSS Negative self	.16 (.03 to .34)	.19 (−.39 to .76)	.34 (.06 to .69)
		PAMR anxious ➔ BCSS Negative others	.17 (.04 to .34)	.14 (−.38 to .65)	.39 (.02 to .81)
		PAMR disorganized ➔ BCSS Negative self	.12 (.01 to .30)	.13 (−.43 to .68)	.40 (.10 to .77)
		PAMR disorganized ➔ BCSS Negative others	.20 (.06 to .40)	.13 (−.38 to .64)	.39 (.01 to .81)
		PAMR avoidant ➔ BCSS Negative self	.05 (−.02 to .15)	.26 (−.30 to .83)	.26 (.01 to .56)
		PAMR avoidant ➔ BCSS Negative others	.08 (−.03 to .23)	.26 (−.26 to .76)	.27 (−.07 to .66)
	CAPE negative frequency	PAMR anxious ➔ BCSS Negative self	.20 (.06 to .35)	.30 (−.09 to .70)	.40 (.07 to .73)
		PAMR anxious ➔ BCSS Negative others	.07 (.01 to .15)	.30 (−.12 to .72)	.41 (.12 to .69)
		PAMR disorganized ➔ BCSS Negative self	.19 (.06 to .37)	.33 (−.07 to .73)	.37 (.06 to .68)
		PAMR disorganized ➔ BCSS Negative others	.08 (.01 to .19)	.37 (−.06 to .80)	.33 (.08 to .60)
		PAMR avoidant ➔ BCSS Negative self	.06 (−.02 to .16)	.38 (−.00 to .77)	.32 (.02 to .62)
		PAMR avoidant ➔ BCSS Negative others	.04 (−.01 to .11)	.50 (.07 to .92)	.21 (−.03 to .46)

*Note*: The effect values shown are unstandardized coefficients.

**TABLE 4:** Indirect, direct and total effects for the relationship between paternal abusive and overcontrolling parenting styles and symptom frequency, serially mediated by insecure attachment style and negative schemas (*n* = 115).

Independent variable	Dependent variable	Mediators	Indirect effect (95% CI)	Direct effect (95% CI)	Total effect (95% CI)
MOPS paternal abuse	CAPE positive frequency	PAMR anxious ➔ BCSS Negative self	.07 (.00 to .17)	.23 (−.13 to .59)	.21 (.05 to .42)
		PAMR anxious ➔ BCSS Negative others	.07 (.01 to .16)	.18 (−.14 to .50)	.26 (.04 to .52)
		PAMR disorganized ➔ BCSS Negative self	.05 (.00 to .13)	.21 (−.14 to .56)	.23 (.06 to .44)
		PAMR disorganized ➔ BCSS Negative others	.09 (.01 to .19)	.18 (−.14 to .50)	.26 (.04 to .50)
		PAMR avoidant ➔ BCSS Negative self	.01 (−.04 to .07)	.26 (−.10 to .62)	.18 (.03 to .38)
		PAMR avoidant ➔ BCSS Negative others	.02 (−.06 to .40)	.23 (−.10 to .55)	.22 (.00 to .45)
	CAPE negative frequency	PAMR anxious ➔ BCSS Negative self	.09 (.01 to .18)	.06 (−.19 to .31)	.27 (.08 to .46)
		PAMR anxious ➔ BCSS Negative others	.03 (.00 to .07)	.10 (−.16 to .36)	.23 (.06 to .42)
		PAMR disorganized ➔ BCSS Negative self	.09 (.01 to .18)	.07 (−.19 to .32)	.26 (.07 to .46)
		PAMR disorganized ➔ BCSS Negative others	.04 (.00 to .09)	.13 (−.14 to .41)	.20 (.03 to .37)
		PAMR avoidant ➔ BCSS Negative self	.02 (−.05 to .08)	.10 (−.15 to .35)	. 23 (.03 to .42)
		PAMR avoidant ➔ BCSS Negative others	.01 (−.02 to .05)	.20 (−.07 to .48)	.12 (−.05 to .29)
MOPS paternal overcontrol	CAPE positive frequency	PAMR anxious ➔ BCSS Negative self	.14 (.02 to .33)	.06 (−.49 to .61)	.42 (.16 to .75)
		PAMR anxious ➔ BCSS Negative others	.14 (.03 to .29)	−.14 (−.64 to .35)	.63 (.26 to 1.04)
		PAMR disorganized ➔ BCSS Negative self	.11 (.01 to .24)	.02 (−.52 to .55)	.47 (.19 to .80)
		PAMR disorganized ➔ BCSS Negative others	.18 (.05 to .35)	−.13 (−.63 to .36)	.62 (.26 to 1.02)
		PAMR avoidant ➔ BCSS Negative self	.02 (−.06 to .10)	.13 (−.42 to .68)	.35 (.11 to .65)
		PAMR avoidant ➔ BCSS Negative others	.04 (−.09 to .16)	−.05 (−.55 to .46)	.53 (.20 to .89)
	CAPE negative frequency	PAMR anxious ➔ BCSS Negative self	.17 (.05 to .33)	−.05 (−.43 to .33)	.52 (.22 to .86)
		PAMR anxious ➔ BCSS Negative others	.06 (.01 to .13)	−.03 (−.44 to .37)	.50 (.19 to .85)
		PAMR disorganized ➔ BCSS Negative self	.18 (.05 to .34)	−.05 (−.43 to .34)	.52 (.22 to .83)
		PAMR disorganized ➔ BCSS Negative others	.07 (.01 to .16)	.04 (−.39 to .46)	.44 (.17 to .72)
		PAMR avoidant ➔ BCSS Negative self	.02 (−.07 to .11)	.05 (−.33 to .43)	.42 (.13 to .74)
		PAMR avoidant ➔ BCSS Negative others	.01 (−.03 to .07)	.21 (−.22 to .63)	.27 (−.00 to .54)

*Note*: The effect values shown are unstandardized coefficients.

When avoidant attachment style was included within the serial mediation models between maternal and paternal abusive parenting style and symptom frequency, there was a non‐significant effect. Similarly, non‐significant results were seen for the indirect effect of maternal and paternal overcontrol on positive and negative symptom frequency, through avoidant attachment and negative self and negative other schemas.

**FIGURE 1:**
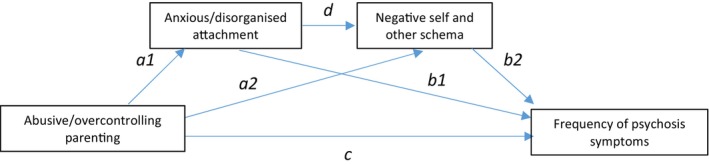
A full serial mediation of anxious/disorganized attachment and negative schema in the relationship between abusive/overcontrolling parenting and symptoms of psychosis.

Therefore, consistent with hypotheses, there was a full serial mediation of both anxious and disorganized attachment and negative self and negative other schemas on the relationship between abusive and overcontrolling parenting style and symptom frequency. However, this was not the case for avoidant attachment, in which a serial mediation effect was not observed. These results were replicated for both maternal and paternal parenting styles. To summarize, the results showed that there was an indirect relationship between abusive or overcontrolling parenting style and positive and negative symptom frequency in people with psychosis, fully mediated by anxious or disorganized attachment style and negative self and negative other schemas. This serial mediation is represented in Figure 1, represented with the a1db2 pathway. However, this relationship was not replicated when applied to an avoidant attachment style.

#### Indifferent parenting and avoidant attachment

**FIGURE 2:**
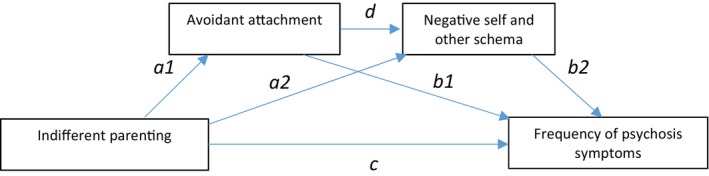
A full serial mediation of avoidant attachment and negative schema in the relationship between indifferent parenting and symptoms of psychosis.

The results of the mediation analyses of avoidant attachment and negative self and other schema in the relationship between indifferent parenting style and symptom frequency are reported in Table 5. Bootstrap confidence intervals showed a significant positive indirect effect of indifferent maternal and paternal parenting style on positive symptom frequency through avoidant attachment and negative self and other schemas. Similarly, there was a significant positive indirect effect of maternal and paternal indifferent parenting on negative symptom frequency, through avoidant attachment and negative self and negative other schemas. The results demonstrate that there was an indirect relationship between indifferent parenting style and positive and negative symptom frequency in people with psychosis, fully mediated by avoidant attachment style and negative self and negative other schemas. This serial mediation is represented in Figure 2, represented with the a1db2 pathway.

**TABLE 5:** Indirect, direct and total effects for the relationship between maternal and paternal indifferent parenting style and symptom frequency, serially mediated by avoidant insecure attachment style and negative schemas (*n* = 115).

Independent variable	Dependent variable	Mediators	Indirect effect (95% CI)	Direct effect (95% CI)	Total effect (95% CI)
MOPS maternal indifference	CAPE positive frequency	PAMR avoidant ➔ BCSS Negative self	.07 (.01 to .16)	.09 (−.31 to .49)	.22 (.04 to .42)
		PAMR avoidant ➔ BCSS Negative others	.10 (.02 to .22)	−.01 (−.38 to .36)	.32 (.07 to .56)
	CAPE negative frequency	PAMR avoidant ➔ BCSS Negative self	.08 (.02 to .17)	.10 (−.18 to .38)	.29 (.10 to .50)
		PAMR avoidant ➔ BCSS Negative others	.05 (.01 to .11)	.13 (−.18 to .44)	.27 (.09 to .46)
MOPS paternal indifference	CAPE positive frequency	PAMR avoidant ➔ BCSS Negative self	.06 (.01 to .13)	−.06 (−.42 to .30)	.29 (.11 to .49)
		PAMR avoidant ➔ BCSS Negative others	.10 (.02 to .20)	−.03 (−.35 to .29)	.25 (.03 to .48)
	CAPE negative frequency	PAMR avoidant ➔ BCSS Negative self	.07 (.01 to .13)	.12 (−.13 to .37)	.34 (.14 to .54)
		PAMR avoidant ➔ BCSS Negative others	.04 (.01 to .10)	.25 (−.02 to .52)	.21 (.06 to .38)

*Note*: The effect values shown are unstandardized coefficients.

## DISCUSSION

This study explored the role of attachment style and schemas in the relationship between parenting style and symptoms of psychosis in adulthood. Although abusive and overcontrolling parenting styles did not independently predict positive or negative symptom frequency in psychosis, this relationship was fully mediated by anxious or disorganized attachment and negative schemas. For individuals who had experienced more indifferent parenting, the relationship between parenting style and psychosis symptoms was mediated by avoidant attachment and negative schemas. The results support the prediction that parenting influences the development of psychosis, building on previous research highlighting a theoretical pathway from childhood trauma, disorganized attachment style, negative schema and paranoia or negative symptoms (Degnan et al., 2022; Humphrey et al., 2022). The current study extends understanding of how different childhood experiences of parenting from primary caregivers lead to child, and later adult, attachment styles. Results suggest there are multiple and different pathways to psychosis in adulthood, which parenting style appears to contribute to.

The results suggest that parents who are abusive and/or overcontrolling are more likely to result in anxious or disorganized attachment styles, leading to negative schemas and a greater frequency of positive and negative symptoms in adulthood. The MOPS conceptualizes abusive parenting through statements about verbal and physical abuse, and the emotional impact of abuse such as ‘made me feel in danger’ and ‘made me feel unsafe’ (Parker et al., 1997). The MOPS differs from other measures of childhood trauma which focus on exposure to specific adverse life events, for example, sexual assault, witness to violence or natural disasters (Goldberg & Freyd, 2006). The MOPS specifically captures the experience of abuse from primary caregivers and feeling unsafe within the family home, but does not consider other traumatic experiences, such as assault by a stranger. Abusive parenting has previously been associated with a disorganized attachment style, which has been considered a response to the contradictory experience of parents who are supposed to provide a place of safety, but instead elicit fear (Main & Solomon, 1986; Pollard et al., 2020). Childhood physical abuse has previously been associated with anxious attachment, which is supported by the results of the current study (Unger & De Luca, 2014). It could be hypothesized that children develop an anxious attachment style as a mechanism for avoiding further abuse. For example, by being hypervigilant to changes in emotion, children may learn to keep caregivers happy. However, it could alternatively be hypothesized that a child with an anxious attachment may be less independent and seek more closeness with caregivers, leading to negative self schemas that justify the abuse in their minds (e.g., ‘I deserve it’). An avoidant attachment style may also be an adaptive response to abuse through the avoidance of interactions and limiting displays of distress to prevent triggering a response from caregivers. In the current study, the link with avoidant attachment was less clear, therefore further research is needed to explore these links.

Parental overcontrol is defined as parenting behaviour that is invasive, assertive and restricts a child's autonomy (Grolnick & Pomerantz, 2009). The MOPS conceptualizes overcontrol through statements about overprotection, overcontrol and criticism from caregivers, and the resulting emotional impact, such as ‘sought to make me feel guilty’. There may be overlap between types of parenting, as abusive parents may also demonstrate overcontrol. This is supported by the current study, as MOPS abuse scores were moderately significantly correlated with overcontrol scores. Therefore, in some cases, overcontrol may be a form of abuse. Conversely, literature suggests that the presence of higher levels of warmth alongside parental overcontrol may improve child mental health outcomes, suggesting that overcontrol may not always be experienced negatively (Fox et al., 2023). Although parental overcontrol has been seen to be associated with poor adolescent adjustment, research suggests it is moderated by parent–child attunement, and the selective use of overcontrol to shield children from danger may protect against poor adjustment (Miller et al., 2018). It is suggested that insecure anxious attachment may develop as a response to caregivers that are insensitive or unresponsive to distress, and therefore, the child has to amplify their distress in order to get their needs met (De Wolff & Van Ijzendoorn, 1997; Pollard et al., 2020). It could be hypothesized that the rigidness of overcontrolling parenting leaves children feeling as though their distress is not heard and so they develop an anxious attachment, to gain a sense of control in an environment in which they have very little control. Parents may display different parenting styles at different times, and thus, individuals may have experienced both overcontrol and abuse from caregivers. This may explain the relationship with disorganized attachment styles, which are also suggested to be related to inconsistent parenting (Pollard et al., 2020).

The final parenting style assessed was indifference or neglect. Parental neglect is defined by instances in which parents do not meet the basic and emotional needs of their child (Tang, 2008). The MOPS indifference subscale taps into neglectful childhood experiences from parents or caregivers through items such as ‘would forget about me’ and ‘left me on my own a lot’, highlighting parenting styles in which the child's needs were not met, aligning with the definition of neglect (Dubowitz et al., 1993; Parker et al., 1997). Individuals who experienced indifferent parenting were more likely to report greater levels of avoidant attachment and negative schemas, associated with a higher frequency of both positive and negative symptoms. The current study adds to understanding of how different parenting styles, which may not specifically be considered ‘big T’ traumas, lead to the development of insecure attachment and negative schemas, which have a negative impact on mental health (Neborsky, 2003).

Although negative self and negative other schemas may develop due to maladaptive parenting experiences, it could be hypothesized that they are further established and reinforced by an individual's response to these experiences, guided by their attachment style. For example, an individual may find it hard to make sense of why they respond in a disorganized manner, leading to the development of negative schemas about their own self‐worth. Previous research suggests that schemas of people with mental health difficulties differ based on their attachment style, and individuals with the same attachment style are likely to endorse the same schemas (Bartholomew & Horowitz, 1991; Mason et al., 2005). Therefore, the style of parenting experienced may lead to the development of a specific attachment style, subsequently influencing the type of negative schematic belief that an individual develops, which may then in turn be linked to the likelihood of different psychosis presentations. For example, paranoia, hallucinations or negative symptoms may be associated with different attachment styles and schema groupings. It could be hypothesized that negative self‐beliefs might drive critical auditory hallucinations and negative others schematic beliefs could foster paranoia.

### Strengths and limitations

Much of the previous literature focuses on the impact of distinct childhood traumas on psychosis, thus the current study benefits from considering more subtle aspects of parenting, which may not be captured by trauma measures, but nevertheless be associated with psychosis. There are, however, limitations to the study which need to be considered when interpreting the results. For example, the internal consistency of the overcontrol subscale of the MOPS was questionable, which must be taken into account when considering the reliability of the results involving this subscale. In terms of the representativeness of the sample, there was a high proportion of females in comparison to males. The sample was also well educated, with almost 70% having a degree or higher‐level qualification. Consequently, the results may be less representative of males and individuals with a lower level of education. Due to the online recruitment strategy, it was also not possible to know where the final sample of participants came from. For example, whether they came from social media websites (e.g., facebook, twitter, Instagram), forum websites or other charities. There are also limitations to the use of a cross‐sectional design within serial mediation analysis, as of the order of mediators is not certain. However, there is good theoretical justification for the order of mediators included within this cross‐sectional study, due to evidence that attachment style forms during early childhood and may influence the development of self and other schema (Humphrey et al., 2022; Li et al., 2023; Simard et al., 2011). Experimental research has also suggested that negative self and other beliefs mediate the relationship between manipulated attachment imagery and non‐clinical paranoia, supporting the order of mediators in this study (Sood et al., 2021). Additionally, although attachment styles are suggested to form in early childhood, there is evidence to indicate that maladaptive schemas are not fully developed until adulthood (Braet et al., 2013; Muris, 2006). The current study focused on the frequency of symptoms rather than distress, which was also measured. Although clinicians are primarily interested in distress resulting from symptoms of psychosis, it was important to first identify a link with symptom presence. Additionally, correlational analyses highlighted a high correlation between symptom frequency and distress. As a result, it would have been redundant to measure both in this study. Future research may consider the relationship with symptom distress in more detail. It must also be acknowledged that the Monte Carlo power analysis suggested a sample of 140 was required to achieve 80% power, whereas the final sample consisted of 132 participants. This limitation must be considered when interpreting any non‐significant results. Although similar results were seen for both maternal and paternal parenting, in the current study, the mother was most often the primary caregiver. In addition to mothers, there were a range of other primary caregivers including fathers, other relatives and foster parents. It is important to note that the included measures did not capture these differences in caregivers, and participants were encouraged to answer the mother and father questions with regards to whomever their two primary caregivers were, if possible. Future measures may benefit from acknowledgment of the variation in family systems, which may have an impact on results. Finally, it is worth highlighting limitations relating to the mediation language used, as ‘full’ mediation suggests all possible mediators and suppressors were measured, with no room for error. For this reason, the terms ‘full’ and ‘partial’ mediation in this study should be interpreted alongside this understanding (Hayes, 2017; Hayes & Rockwood, 2016).

### Clinical implications

The findings highlight the benefit of considering more subtle childhood experiences of parenting, attachment styles and negative schema in addition to specific traumas when developing psychological formulations with individuals who have experiences of psychosis. A focus on identifying key maladaptive schemas may help to raise awareness of their influence and develop strategies to strengthen more adaptive schemas. Schema therapy may be helpful to consider for people with psychosis but the benefits of this approach with this group need to be tested through randomized controlled trials. Similarly, CBT‐p often involves a focus on an individuals' core beliefs. Nonetheless, keeping attachment theory and relational models in mind when working with people with psychosis may be important in therapy. For example, attachment imagery or attachment security priming involves visualizing memories associated with secure attachments, which is suggested to reduce symptoms of paranoia, anxiety and negative affect, instead increasing positive affect, in people with non‐clinical paranoia (Newman‐Taylor et al., 2021). Symptom reduction has been seen to be mediated by cognitive fusion and negative self and other beliefs (Sood et al., 2021). Using secure attachment imagery with individuals with psychosis may have an influence on negative schemas and subsequent symptom reduction.

The results of the study highlight a need to identify maladaptive parenting styles and the development of insecure attachment as soon as possible in early childhood. Therefore, preschool age services such as health visiting would benefit from staff support and service development to ensure professionals are able to identify maladaptive parenting and the development of early insecure attachments, so that timely intervention can be sought. Funding for parenting interventions may support parents to make more adaptive parenting choices, which may help to avoid the development of insecure attachment. For example, Circle of Security groups have been found to have a beneficial effect on the attachment styles of preschool and early school‐aged children and their parents caregiving patterns (Hoffman et al., 2006). Triple P and Incredible years programs may also help to improve parenting style; however, more research is needed in relation to their impact on attachment (Sanders et al., 2020; Webster‐Stratton, 2005). The study findings were replicated for both parents, highlighting the importance of supporting fathers in their interactions with their children. Perinatal mental health services are usually offered to mothers only; however, fathers and other caregiver interactions may also have a vital impact on childhood attachment, schemas and later psychosis. Additionally, there may be a relationship between maternal and paternal parenting styles, especially in cases where a child is co‐parented. Future research may benefit from considering both parents together, whether that is mother and father, or involves another parenting combination, such as grandparent and parent.

### Future research

This research highlights priorities for future research in the areas of both child development and intervention testing. For example, large scale general population longitudinal studies following participants from childhood into adulthood, considering parenting styles, development of early attachment, subsequent schema development and later mental health, including psychosis. Using a longitudinal method would increase the confidence placed on the order of mediators, providing confirmation for the current cross‐sectional study and further develop theory regarding predictors of psychosis. Furthermore, experimental research could be utilized to investigate how specific attachment and schema techniques might be used in therapy to influence symptoms in psychosis. For example, through manipulation of attachment imagery between groups. Although avoidant attachment was more associated with indifferent parenting than the other insecure attachment styles, it was unclear from the current study what differences led to either anxious or disorganized attachment styles. Further research to increase understanding of the development of anxious and disorganized attachment styles, and the subtle types of parenting that might be more associated with each may help to provide a richer understanding of the predictions outlined in this study. For example, using measures of childhood trauma such as the Childhood Trauma Questionnaire (CTQ), to determine whether the type of abuse is important in the subsequent attachment style which develops (Bernstein et al., 1998). Detailed parenting measures that are aligned with the schema therapy model may also facilitate this understanding, such as the Young Parenting Inventory (YPI) or the Positive Parenting Schema Inventory (PPSI) (Louis et al., 2020; Young et al., 2003). Additionally, further research might consider a more detailed exploration of individual schemas and their relation to different parenting and attachment styles, and to specific symptoms of psychosis. For example, abusive parenting may be linked with specific negative schemas, such as that other people are dangerous, which may be associated with paranoia. More comprehensive measures of schema such as the Young Schema Questionnaire (YSQ) may be useful to decipher participant's individual schemas (Young, 1998).

Additionally, future research would benefit from a focus on schema change as a result of psychological interventions in people with experiences of psychosis. Cognitive Behaviour Therapy for psychosis (CBT‐p) is a recommended first‐line psychological intervention, alongside antipsychotic medication, for treating first episode and longstanding psychosis within both UK and international guidance (Addington et al., 2017; Early Psychosis Guidelines Writing Group, 2010; Keepers et al., 2020; National Institute for Health and Care Excellence, 2014). Therefore, research should determine whether CBT is effective at targeting and shifting maladaptive schemas in people with psychosis, and whether this leads to subsequent symptom reduction. As schema therapy is designed to place a focus on identifying and changing negative schemas, further research into this mode of therapy for people with experiences of psychosis would also be beneficial, as evidence is currently limited (Taylor et al., 2017; Young et al., 2003). Further development of evidence‐based parenting interventions would help to understand whether current interventions are effective at forming more secure childhood attachment styles and subsequently, endorsement of fewer maladaptive schemas. Tailoring parent–infant interventions to foster the development of secure attachment and positive self and other schemas may have an important effect on children's subsequent mental health and potentially reduce the likelihood and/or severity of psychosis in adulthood.

## CONCLUSION

Overall, the findings provide evidence for the relevance of parenting, attachment styles and the resulting schema development within psychosis theory. Longitudinal research is now needed to provide a more rigorous test of these relationships. While we await the results of this longitudinal research, there is enough evidence to justify the consideration of parenting experiences, attachment styles and schema in assessment, formulation and therapy for people experiencing psychosis. The results from this study also support the use and further development of parenting and attachment interventions for families.

## AUTHOR CONTRIBUTIONS

**Nadia Akers:** Conceptualization; investigation; formal analysis. **Christopher D. J. Taylor:** Conceptualization; supervision. **Katherine Berry:** Conceptualization; supervision.

## CONFLICT OF INTEREST STATEMENT

Authors have no conflict of interest to declare.

## Data Availability

The data that support the findings of this study are available on request from the corresponding author. The data are not publicly available due to privacy or ethical restrictions.

## APPENDIX A COVARIATE ANALYSES

**TABLE A1:** Indirect, direct and total effects for the relationship between maternal abusive and overcontrolling parenting styles and symptom frequency, serially mediated by insecure attachment style and negative schemas, with employment and ethnicity as covariates (*n* = 115).

Independent variable	Dependent variable	Mediators	Indirect effect (95% CI)	Direct effect (95% CI)	Total effect (95% CI)
MOPS maternal abuse	CAPE positive frequency	PAMR anxious → BCSS Negative self	.10 (.02 to .24)	.11 (−.28 to .51)	.33 (.09 to .62)
		PAMR anxious → BCSS Negative others	.09 (.01 to .20)	.02 (−.35 to .39)	.34 (.10 to .64)
		PAMR disorganized → BCSS Negative self	.08 (.01 to .18)	.08 (−.30 to .47)	.27 (.06 to .54)
		PAMR disorganized → BCSS Negative others	.11 (.02 to .25)	.03 (−.33 to .39)	.32 (.08 to .61)
		PAMR avoidant → BCSS Negative self	.04 (−.01 to .12)	.14 (−.25 to .54)	.21 (.02 to .47)
		PAMR avoidant → BCSS Negative others	.06 (−.02 to .16)	.09 (−.28 to .46)	.26 (.04 to .54)
	CAPE negative frequency	PAMR anxious → BCSS Negative self	.11 (.02 to .23)	−.04 (−.32 to .23)	.28 (.05 to .53)
		PAMR anxious → BCSS Negative others	.04 (.01 to .09)	−.05 (−.34 to .24)	.29 (.10 to .50)
		PAMR disorganized → BCSS Negative self	.11 (.02 to .21)	−.03 (−.31 to .25)	.27 (.05 to .50)
		PAMR disorganized → BCSS Negative others	.05 (.00 to .12)	−.01 (−.31 to .30)	.24 (.07 to .41)
		PAMR avoidant → BCSS Negative self	.04 (−.01 to .12)	−.01 (−.28 to .27)	.25 (.04 to .46)
		PAMR avoidant → BCSS Negative others	.03 (−.01 to .08)	.05 (−.25 to .36)	.19 (.23 to .36)
MOPS maternal overcontrol	CAPE positive frequency	PAMR anxious → BCSS Negative self	.18 (.04 to .37)	.10 (−.48 to .68)	.39 (.10 to .77)
		PAMR anxious → BCSS Negative others	.16 (.04 to .34)	.08 (−.45 to .61)	.37 (.00 to .79)
		PAMR disorganized → BCSS Negative self	.13 (.02 to .32)	.00 (−.56 to .56)	.45 (.13 to .83)
		PAMR disorganized → BCSS Negative others	.20 (.05 to .41)	.07 (−.45 to .59)	.38 (.00 to .79)
		PAMR avoidant → BCSS Negative self	.06 (−.02 to .16)	.13 (−.44 to .70)	.32 (.06 to .64)
		PAMR avoidant → BCSS Negative others	.08 (−.03 to .22)	.22 (−.31 to .74)	.24 (−.10 to .62)
	CAPE negative frequency	PAMR anxious → BCSS Negative self	.19 (.06 to .34)	.36 (−.04 to .75)	.42 (.12 to .73)
		PAMR anxious → BCSS Negative others	.07 (.01 to .15)	.39 (−.02 to .80)	.39 (.12 to .65)
		PAMR disorganized → BCSS Negative self	.18 (.06 to .34)	.38 (−.02 to .78)	.40 (.11 to .68)
		PAMR disorganized → BCSS Negative others	.08 (.01 to .19)	.46 (−.03 to .88)	.32 (.08 to .57)
		PAMR avoidant → BCSS Negative self	.05 (−.02 to .15)	.44 (.05 to .82)	.34 (.07 to .61)
		PAMR avoidant → BCSS Negative others	.03 (−.01 to .10)	.58 (.17 to 1.00)	.19 (−.03 to .42)

*Note*: The values shown are unstandardized coefficients.

**TABLE A2:** Indirect, direct and total effects for the relationship between paternal abusive and overcontrolling parenting styles and symptom frequency, serially mediated by insecure attachment style and negative schemas, with employment and ethnicity as covariates (*n* = 115).

Independent variable	Dependent variable	Mediators	Indirect effect (95% CI)	Direct effect (95% CI)	Total effect (95% CI)
MOPS paternal abuse	CAPE positive frequency	PAMR anxious → BCSS Negative self	.07 (.00 to .17)	.21 (−.14 to .57)	.21 (.04 to .43)
		PAMR anxious → BCSS Negative others	.07 (.01 to .15)	.18 (−.14 to .50)	.24 (.02 to .45)
		PAMR disorganized → BCSS Negative self	.05 (.00 to .13)	.19 (−.15 to .54)	.23 (.05 to .44)
		PAMR disorganized → BCSS Negative others	.09 (.01 to .19)	.18 (−.14 to .50)	.24 (.02 to .49)
		PAMR avoidant → BCSS Negative self	.01 (−.05 to .07)	.24 (−.12 to .60)	.18 (.02 to .39)
		PAMR avoidant → BCSS Negative others	.02 (−.06 to .10)	.23 (−.10 to .55)	.20 (.02 to .44)
	CAPE negative frequency	PAMR anxious → BCSS Negative self	.08 (.01 to .16)	.05 (−.19 to .30)	.24 (.06 to .43)
		PAMR anxious → BCSS Negative others	.03 (.00 to .07)	.09 (−.17 to .35)	.20 (.04 to .38)
		PAMR disorganized → BCSS Negative self	.08 (.01 to .16)	.06 (−.20 to .31)	.24 (.06 to .43)
		PAMR disorganized → BCSS Negative others	.03 (.00 to .08)	.12 (−.15 to .39)	.18 (.02 to .34)
		PAMR avoidant → BCSS Negative self	.01 (−.05 to .07)	.09 (−.15 to .34)	. 20 (.02 to .38)
		PAMR avoidant → BCSS Negative others	.01 (−.02 to .05)	.19 (−.08 to .46)	.02 (−.06 to .26)
MOPS paternal overcontrol	CAPE positive frequency	PAMR anxious → BCSS Negative self	.14 (.02 to .33)	.02 (−.53 to .58)	.42 (.13 to .75)
		PAMR anxious → BCSS Negative others	.13 (.02 to .28)	−.13 (−.64 to .38)	.57 (.19 to 1.00)
		PAMR disorganized → BCSS Negative self	.11 (.01 to .26)	−.04 (−.58 to .51)	.47 (.17 to .82)
		PAMR disorganized → BCSS Negative others	.17 (.04 to .36)	−.13 (−.63 to .38)	.57 (.20 to 1.00)
		PAMR avoidant → BCSS negative self	.01 (−.09 to .10)	.09 (−.47 to .65)	.35 (.09 to .67)
		PAMR avoidant → BCSS Negative others	.02 (−.11 to .13)	−.03 (−.54 to .48)	.47 (.14 to .85)
	CAPE negative frequency	PAMR anxious → BCSS Negative self	.15 (.03 to .31)	−.13 (−.50 to .26)	.47 (.18 to .79)
		PAMR anxious → BCSS Negative others	.05 (.01 to .12)	−.09 (−.50 to .31)	.44 (.14 to .78)
		PAMR disorganized → BCSS Negative self	.16 (.04 to .32)	−.13 (−.51 to .26)	.48 (.18 to .79)
		PAMR disorganized → BCSS Negative others	.07 (.01 to .15)	.05 (−.47 to .37)	.39 (.13 to .67)
		PAMR avoidant → BCSS Negative self	.01 (−.09 to .10)	−.03 (−.41 to .36)	.37 (.07 to .67)
		PAMR avoidant → BCSS Negative others	.01 (−.04 to .06)	.13 (−.29 to .56)	.21 (−.04 to .47)

*Note*: The values shown are unstandardized coefficients.

**TABLE A3:** Indirect, direct and total effects for the relationship between maternal and paternal indifferent parenting style and symptom frequency, serially mediated by avoidant insecure attachment style and negative schemas, with employment and ethnicity as covariates (*n* = 115).

Independent variable	Dependent variable	Mediators	Indirect effect (95% CI)	Direct effect (95% CI)	Total effect (95% CI)
MOPS maternal indifference	CAPE positive frequency	PAMR avoidant → BCSS Negative self	.07 (.02 to .17)	.11 (−.29 to .51)	.21 (.03 to .42)
		PAMR avoidant → BCSS Negative others	.10 (.02 to .22)	−.00 (−.37 to .37)	.32 (.08 to .56)
	CAPE negative frequency	PAMR avoidant → BCSS Negative self	.07 (.02 to .15)	.11 (−.17 to .38)	.26 (.08 to .47)
		PAMR avoidant → BCSS Negative others	.04 (.01 to .10)	.12 (−.19 to .43)	.25 (.09 to .44)
MOPS paternal indifference	CAPE positive frequency	PAMR avoidant → BCSS Negative self	.06 (.01 to .14)	−.02 (−.38 to .35)	.26 (.09 to .46)
		PAMR avoidant → BCSS Negative others	.09 (.02 to .19)	−.01 (−.34 to .32)	.25 (.04 to .48)
	CAPE negative frequency	PAMR avoidant → BCSS Negative self	.06 (.01 to .13)	.09 (−.16 to .34)	.30 (.12 to .48)
		PAMR avoidant → BCSS Negative others	.04 (.01 to .09)	.18 (−.09 to .45)	.20 (.06 to .36)

*Note*: The values shown are unstandardized coefficients.
